# Draft Genome Sequences of *Psychrobacter* Strains JCM 18900, JCM 18901, JCM 18902, and JCM 18903, Isolated Preferentially from Frozen Aquatic Organisms

**DOI:** 10.1128/genomeA.00280-14

**Published:** 2014-04-03

**Authors:** Toshiaki Kudo, Akihiro Kidera, Muneaki Kida, Ayumi Kawauchi, Ryo Shimizu, Tomomi Nakahara, Xiaochi Zhang, Akinori Yamada, Masao Amano, Yuki Hamada, Shigeto Taniyama, Osamu Arakawa, Asami Yoshida, Kenshiro Oshima, Wataru Suda, Hirokazu Kuwahara, Yuichi Nogi, Keiko Kitamura, Masahiro Yuki, Toshiya Iida, Shigeharu Moriya, Tetsushi Inoue, Yuichi Hongoh, Masahira Hattori, Moriya Ohkuma

**Affiliations:** a Graduate School of Fisheries Science and Environmental Studies, Nagasaki University, Nagasaki, Japan; b Graduate School of Science and Technology, Nagasaki University, Nagasaki, Japan; c Center for Omics and Bioinformatics, Graduate School of Frontier Sciences, the University of Tokyo, Kashiwa, Japan; d Institute of Biogeosciences, Japan Agency for Marine-Earth Science and Technology (JAMSTEC), Yokosuka, Japan; e Department of Biological Sciences, Graduate School of Bioscience and Biotechnology, Tokyo Institute of Technology, Tokyo, Japan; f Japan Collection of Microorganisms/Microbe Division, RIKEN BioResource Center, Tsukuba, Japan; g Biomass Research Platform Team, RIKEN Biomass Engineering Program Cooperation Division, RIKEN Center for Sustainable Resource Science, Tsukuba, Japan; h Research Cluster for Innovation, RIKEN, and Graduate School of Nanobioscience, Yokohama City University, Yokohama, Japan

## Abstract

Four *Psychrobacter* strains, JCM 18900, JCM 18901, JCM 18902, and JCM 18903, related to either *Psychrobacter nivimaris* or *Psychrobacter cibarius*, were isolated from frozen marine animals. The genome information of these four strains will be useful for studies of their physiology and adaptation properties to frozen conditions.

## GENOME ANNOUNCEMENT

The genus *Psychrobacter* comprises Gram-negative, psychrotolerant, aerobic, and nonmotile bacteria (1). They are able to grow at 5°C. They are halotolerant and grow in the presence of ≥6.5% NaCl. *Psychrobacter* species have been isolated from various cold habitats (1).

We found that *Psychrobacter* strains related to *Psychrobacter nivimaris* (2) and *Psychrobacter cibarius* (3) were preferentially isolated from various frozen marine animals, such as the finless porpoise *Neophocaena phocaenoides*, the scavenging gastropod *Nassarius* (*Alectrion*) *glans*, the silver jewfish *Pennahia argentata*, and the skipjack tuna *Katsuwonus pelamis. Psychrobacter* strains JCM 18900, JCM 18901, JCM 18902, and JCM 18903 (originally described as strains B120, B216, B295, and B296, respectively) were isolated from intestinal samples of the frozen finless porpoise *N. phocaenoides* and stored for approximately 1 year at −20°C.

The genomes of *Psychrobacter* strains JCM 18900, JCM 18901, JCM 18902, and JCM 18903 were sequenced using an Ion Torrent PGM system. The sequence reads of 769,319 for JCM 18900, 537,775 for JCM 18901, 610,006 for JCM 18902, and 643,259 for JCM 18903 were assembled using Newbler version 2.8 (Roche) into 35, 58, 33, and 56 contigs with *N*_50_ lengths of 249,741, 117,508, 264,897, and 162,339 bp, respectively. These assemblies resulted in draft genome sequences of 3,272,645 bp for JCM 18900, 3,145,827 bp for JCM 18901, 3,274,327 bp for JCM 18902, and 3,427,960 bp for JCM 18903, with 51×, 38×, 36×, and 36× redundancies and G+C contents of 42.7, 42.7, 42.9, and 42.7%, respectively. A total of 2,893, 2,875, 2,798, and 3,004 protein-coding genes and 40, 44, 45, and 45 RNA-coding sequences for JCM 18900, JCM 18901, JCM 18902, and JCM 18903, respectively, were identified using the RAST server (4).

Phylogenetic analysis based on 16S rRNA gene sequence comparisons revealed that strains JCM 18900 and JCM 18901 are closely related to the type strain of *P. nivimaris* (sequence identity, 99%), while strains JCM 18902 and JCM 18903 are closely related to the type strain of *P. cibarius* (sequence identity, 100%). These four strains possess genes for a wax ester synthase, production of capsular type extracellular polymeric substances, and three cold shock proteins, which are important factors for adaptation to the cold (5). The genome information of these four strains will be useful for studies of their physiology and adaptation properties to frozen conditions.

### Nucleotide sequence accession numbers.

The genome sequences of *Psychrobacter* strains JCM 18900, JCM 18901, JCM 18902, and JCM 18903 have been deposited at DDBJ/EMBL/GenBank under accession no. BAWG01000001 to BAWG01000035, BAWH01000001 to BAWH01000058, BAWI01000001 to BAWI01000033, and BAWJ01000001 to BAWJ01000056, respectively.
